# Tick Histamine Release Factor Is Critical for *Ixodes scapularis* Engorgement and Transmission of the Lyme Disease Agent

**DOI:** 10.1371/journal.ppat.1001205

**Published:** 2010-11-24

**Authors:** Jianfeng Dai, Sukanya Narasimhan, Lili Zhang, Lei Liu, Penghua Wang, Erol Fikrig

**Affiliations:** 1 Section of Infectious Diseases, Department of Internal Medicine, Yale University School of Medicine, New Haven, Connecticut, United States of America; 2 Howard Hughes Medical Institute, Chevy Chase, Maryland, United States of America; Medical College of Wisconsin, United States of America

## Abstract

Ticks are distributed worldwide and affect human and animal health by transmitting diverse infectious agents. Effective vaccines against most tick-borne pathogens are not currently available. In this study, we characterized a tick histamine release factor (tHRF) from *Ixodes scapularis* and addressed the vaccine potential of this antigen in the context of tick engorgement and *B. burgdorferi* transmission. Results from western blotting and quantitative Reverse Transcription-PCR showed that tHRF is secreted in tick saliva, and upregulated in *Borrelia burgdorferi*-infected ticks. Further, the expression of tHRF was coincident with the rapid feeding phase of the tick, suggesting a role for tHRF in tick engorgement and concomitantly, for efficient *B. burgdorferi* transmission. Silencing tHRF by RNA interference (RNAi) significantly impaired tick feeding and decreased *B. burgdorferi* burden in mice. Interfering with tHRF by actively immunizing mice with recombinant tHRF, or passively transferring tHRF antiserum, also markedly reduced the efficiency of tick feeding and *B. burgdorferi* burden in mice. Recombinant tHRF was able to bind to host basophils and stimulate histamine release. Therefore, we speculate that tHRF might function *in vivo* to modulate vascular permeability and increase blood flow to the tick bite-site, facilitating tick engorgement. These findings suggest that blocking tHRF might offer a viable strategy to complement ongoing efforts to develop vaccines to block tick feeding and transmission of tick-borne pathogens.

## Author Summary

Ticks are distributed worldwide and affect human and animal health by transmitting diverse infectious agents. Safe and effective vaccines against most tick-borne pathogens are not currently available. Typical vaccines target microbes directly, using extracts of the organism, or recombinant antigens as the immunogen; the transmission of tick-borne pathogens can also theoretically be prevented by interfering with the ability of ticks to feed on a mammalian host. In this study, we have characterized a putative histamine release factor (tHRF) from *I. scapularis* ticks, the predominant vector of *B. burgdorferi*, the agent of Lyme disease in North America. Our results suggested that tHRF is presented in tick saliva and critical for tick feeding; blocking tHRF markedly reduced the efficiency of tick feeding, and reduced the *B. burgdorferi* burden in mice. This finding provides novel insights into the molecular mechanisms of tick feeding and provides a potential vaccine target to block tick feeding and pathogen transmission.

## Introduction

Ticks are distributed worldwide and affect human and animal health by transmitting diverse infectious agents. Ticks are considered to be second to mosquitoes as major vectors of human diseases [1], [2]. For example, Ixodes spp., transmit *Borrelia burgdorferi* (the Lyme disease agent), *Anaplasma phagocytophilum* (the cause of human granulocytic anaplasmosis), *Babesia microti*, and tick-borne encephalitis virus (TBEV), among other pathogens [1], [3].

Effective vaccines against most tick-borne pathogens are not currently available and there is an urgent need for the control of ticks and their associated pathogens [4]. Typical vaccines target microbes directly, using extracts of the organism, or recombinant antigens as the immunogen. For example, *B. burgdorferi* outer surface protein A has been extensively studied and resulted in an Federal Drug Administration-approved vaccine that was commercially available from 1998 until 2002 [5], [6]. Currently one vaccine is approved and available for protection against a tick-borne pathogen – TBEV, which is transmitted by *I. ricinus* in Northern Europe and Asia [4].

The transmission of tick-borne pathogens can also theoretically be prevented by interfering with the ability of ticks to feed on a mammalian host [7]. A pilot study by Allen and Humphreys several decades ago, suggested that vaccines based on tick gut antigens successfully reduced *Boophilus* engorgement on cattle [8]. Recently, immunization of guinea pigs with a tick salivary antigen, sialostatin L2, diminished the capacity of *Ixodes scapularis* nymphs to feed [9]. While reducing the ability of tick feeding, tick-based vaccines may have another equally important impact – to decrease the chance of transmission of tick-borne pathogens [10]. Immunization of cattle with Bm86 vaccines resulted in lower infestations as well as decreased incidence of babesiosis and *Anaplasma marginale* infection in some regions [1], [7]. Repeated exposure of guinea pigs to ticks causes acquired resistance of the animals to subsequent tick bites [11], [12], and this development of “tick-immunity” can decrease tick-transmitted *B. burgdorferi* infection [13]. *B. burgdorferi* need to replicate within the ticks during blood feeding and are transmitted to the host after 36–48 h of tick attachment [2], [14], [15], [16]. Thus, impairing *I. scapularis* feeding could be another useful strategy to reduce *B. burgdorferi* transmission.

Tick saliva contains molecules that are important for formation and maintenance of the feeding cavity in the host dermis, as well as the transmission of tick-borne pathogens [17], [18]. These activities include anti-hemostatic, anti-inflammatory and immunomodulatory effects, among others [17], [19]. Histamine binding proteins are well characterized and suggested to be important to neutralize the inflammatory effect of histamine, which is secreted by host immune cells at the tick feeding site and critical for Ixodes ticks to successfully attach to the host [20], [21]. Interestingly, *Dermacentor variabilis* ticks also express a protein in their saliva, which shares high homology with mammalian histamine release factor [22]. Given the deleterious effects of histamine on tick physiology, it is very surprising that ticks encode a histamine release protein that would presumably stimulate histamine secretion. The role of the tick histamine release factor *in vivo* during tick feeding is not understood and warrants detailed examination.

Tick feeding can be divided into a series of 9 stages [2] beginning with host seeking, and culminating in engorgement on the host followed by detachment and dropping off the host. A feeding lesion is established about 24 h post attachment, and during this early phase of feeding there is minimal blood intake. Blood meal ingestion begins slowly around 48 h post tick attachment followed by rapid feeding to repletion around 72 h–96 h post tick attachment (late stage). While it is recognized that the *I. scapularis* salivary gland proteome changes during these early and late phases of feeding [13], a molecular understanding of these events remains to be elucidated.

In this study, we have characterized a putative histamine release factor from *I. scapularis*, the predominant vector of *B. burgdorferi*, the agent of Lyme disease in North America. We invoke a pivotal role for *I. scapularis* HRF during the rapid phase of tick feeding, and address the vaccine potential of this antigen in the context of tick engorgement and *B. burgdorferi* transmission.

## Results

### An *I. scapularis* histamine release factor (tHRF) is up-regulated in *B. burgdorferi*-infected ticks

**Figure 1:**
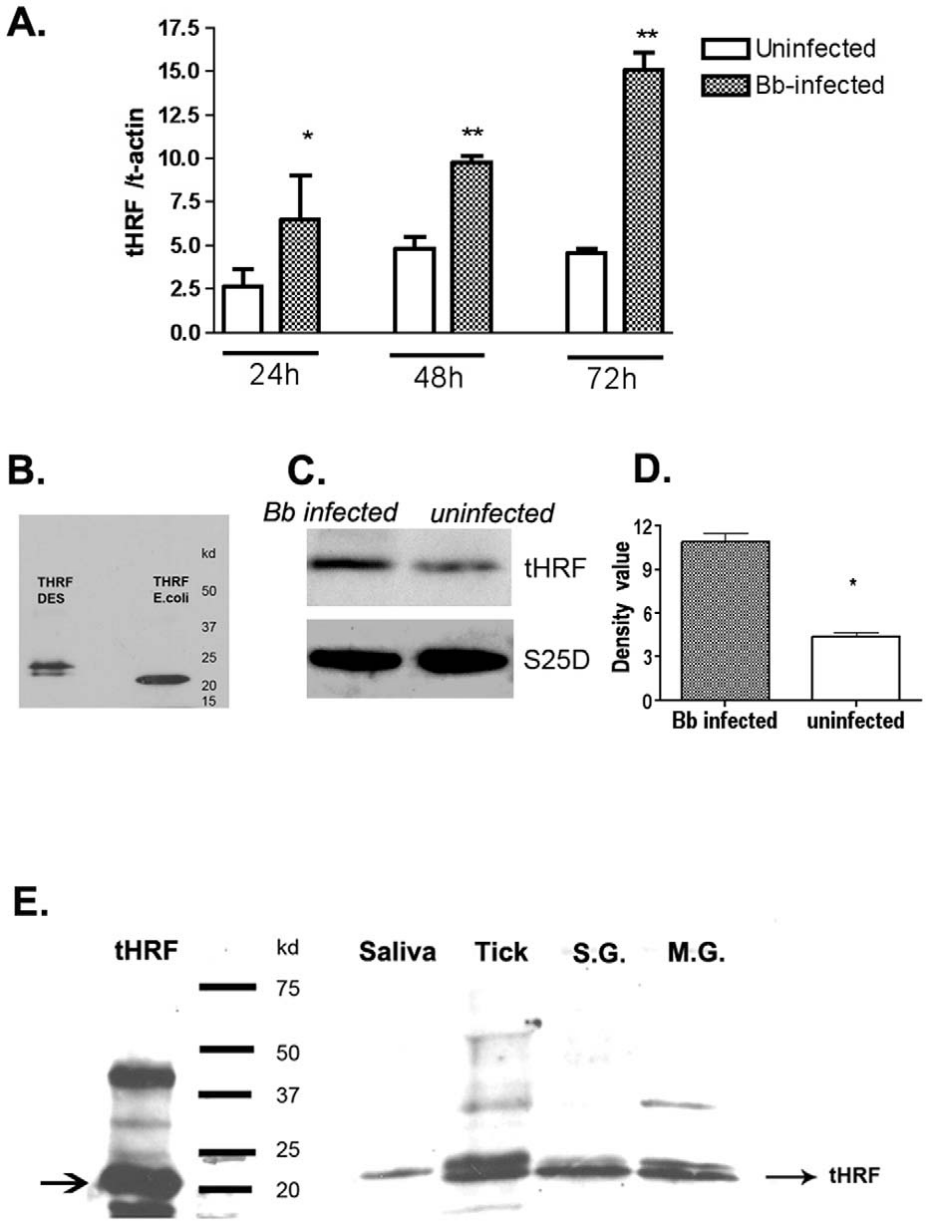
Tick histamine release factor (tHRF) was up-regulated in *B. burgdorferi*- infected ticks. (A) The expression of *tHRF* in *B. burgdorferi*-infected or uninfected ticks during *I. scapularis* feeding. Results are expressed as the mean ± the SEM (*p<0.05; **p<0.01). (B) tHRF-antiserum recognizes recombinant tHRF generated in *Escherichia coli* (tHRF *E.coli*) or Drosophila S2 cells (tHRF DES). (C–D) The up-regulation of tHRF protein in *B. burgdorferi*-infected ticks. (*p<0.05) (E) The detection of tHRF in adult tick saliva (Saliva), nymphal tick salivary glands (S.G.), midgut (M.G.) and whole ticks (Tick).

To identify tick proteins that may be utilized by *B. burgdorferi* to facilitate transmission, 2-dimensional fluorescence difference gel electrophoresis (DIGE) was performed using extracts from *B. burgdorferi*-infected, and uninfected, *I. scapularis* salivary glands. Seventeen differentially expressed proteins (5-fold or more expression levels in *Borrelia*-infected salivary glands) were selected for mass spectrometric analysis, and 4 *I. scapularis* proteins were unambiguously identified with significant MASCOT scores (*p*<0.05) of 79 (Table S1). In this study, we characterize one of the most highly induced proteins, named tHRF because it shares high homology with a murine histamine release factor (57.1% similarity and 40.1% identity at amino acid level). *tHRF* mRNA levels were induced during *I. scapularis* engorgement, and significantly higher in *B. burgdorferi*-infected, than in uninfected, ticks (*p*<0.01). Immunoblots using tHRF antiserum further demonstrated a ∼2.5 fold up-regulation of tHRF in *B. burgdorferi*-infected ticks (Figure 1, A, C–D). tHRF was present in tick saliva, as well as in the salivary glands and midgut, indicating that it is a secreted protein (Figure 1E).

### Silencing *tHRF* impairs tick feeding and *Borrelia* transmission

**Figure 2:**
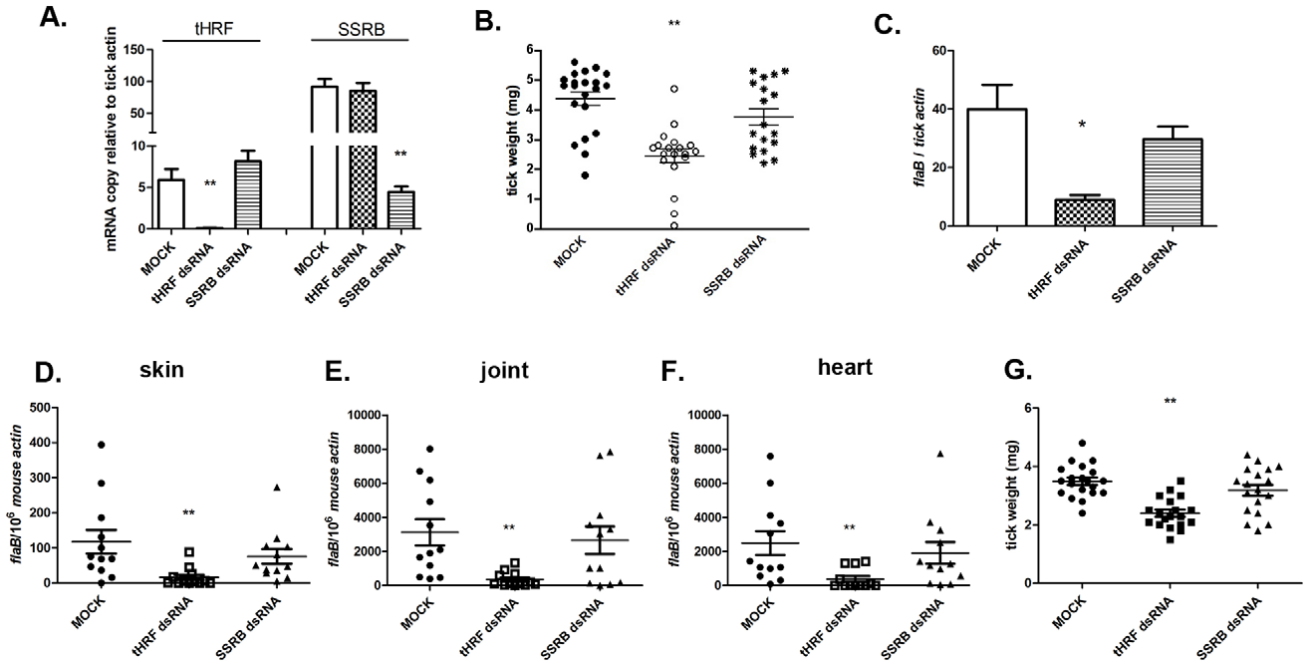
Silencing *tHRF* impairs tick feeding and *B. burgdorferi* transmission. (A) *tHRF* and *SSRB* (another tick gene, as a control) were silenced efficiently and specifically in nympal tick salivary gland by micro-injection of dsRNA. Results are expressed as the mean ± the SEM. (B) The weight of *B. burgdorferi*-infected ticks after feeding on naive mice. (C) The *B. burgdorferi* burden in whole ticks at 72 h post tick-engorgement. Spirochete burden in (D) murine skin at day 7, (E) joints and (F) heart at day 21 post-infection. (G) The weight of uninfected ticks after feeding on naive mice. Horizontal lines and bars represent the mean values ± the SEM. * *p*<0.05 and ** *p*<0.01 when compared with MOCK controls. Results are pooled from 3 independent experiments.

To analyze the potential role of tHRF in tick feeding, and also during *B. burgdorferi* transmission, *tHRF*-deficient *I. scapularis* nymphs were generated by RNA interference (RNAi). Buffer-injected (MOCK), *SSRB* (another tick gene- Single Sequence Receptor Beta- found in our 2DIGE list, used as a control) or *tHRF* double-stranded RNA (dsRNA)-injected *B. burgdorferi*-infected nymphs were allowed to engorge on mice. The silencing of *tHRF* and *SSRB* were confirmed by quantitative RT-PCR (Figure 2A). After 3 days, the weighs of *tHRF*-deficient ticks were significantly lower than control ticks (Figure 2B). Q-PCR revealed a decrease in spirochete levels in *tHRF*-deficient ticks, as well as in the skin of mice that were fed upon by *tHRF*-deficient ticks (Figure 2, C and D). At 3 weeks, when spirochetes have disseminated to diverse organs, the *B. burgdorferi* burden in the heart and joints was also lower in mice infected by *tHRF*-deficient ticks, compared to that in mice infected with control ticks (Figure 2, E and F).

To further show that *tHRF* directly influences tick feeding, an RNAi study was performed with nymphs that were not infected with *B. burgdorferi*. Consistent with the results using *B. burgdorferi*-infected ticks, the tick weight was significantly decreased in *tHRF*-dsRNA-treated uninfected *I. scapularis* after feeding (Figure 2G). These data show that tHRF is critical for tick feeding, regardless of whether *B. burgdorferi* are present within ticks.

### tHRF antiserum prevents efficient tick feeding and *Borrelia* transmission

**Figure 3 ppat-1001205-g003:**
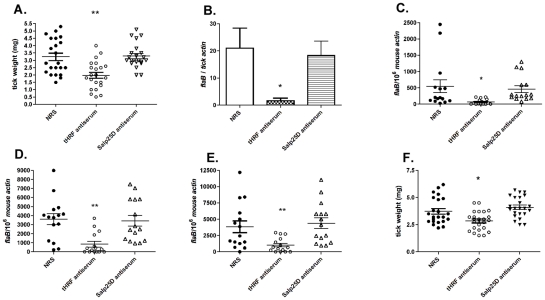
tHRF antiserum interferes with tick feeding and *B. burgdorferi* transmission. (A) Weights of *B. burgdorferi*-infected tick after feeding on tHRF antiserum-immunized, or control, mice. (NRS: normal rabbit serum; rabbit anti-Salp25D antiserum, both used as controls) (B) Spirochete burden in whole *I. scapularis* nymphs after feeding on tHRF antiserum-immunized, or control, mice. (C) *B. burgdorferi* burden in murine skin at day 7 post-tick feeding. Spirochete burden in the (D) joints and (E) heart at day 21 post-tick feeding. (F) Weights of uninfected ticks after feeding on tHRF antiserum-immunized, or control, mice. Horizontal lines and bars represent the mean values ± the SEM. * *p*<0.05 and ** *p*<0.01, when compared with the NRS controls. Results are a composite of independent experiments.

To further examine the importance of tHRF during tick feeding, and its influence on *B. burgdorferi* transmission, a passive immunization study was performed in naive mice. Groups of 5 mice were administered 200 µl of tHRF antiserum, or control sera (normal rabbit serum or Salp25D antiserum; Salp25D is a tick salivary protein that does not influence tick feeding [23]). One day later, 6 *B. burgdorferi*-infected ticks were placed on each mouse and tick weights were assessed after 3 days of feeding. Ticks engorging on tHRF antiserum-treated mice weighed significantly less than ticks that fed on control mice (Figure 3A). The spirochete burden in ticks was also substantially lower in *I. scapularis* that fed on tHRF antiserum-immunized mice (Figure 3B). *B. burgdorferi* burden was also markedly reduced in tHRF antiserum-immunized mice. The spirochete load in murine skin at day 7 post-infection and in joints and hearts at 3 weeks post-infection was markedly lower in tHRF antiserum-immunized group compared to control serum immunized group (Figure 3, C–E). About 20–27% of the tHRF antiserum-immunized mice (N = 15) were fully protected (based on the absence of a detectable *flaB* signal in Q-PCR), while 100% of the control animals were infected (N = 30) (Figure 3, C–E).

Uninfected *I. scapularis* nymphs also fed less efficiently on tHRF antiserum-treated mice (Figure 3F) as seen by decreased engorgement weights compared to ticks fed on control antiserum-treated mice.

**Figure 4:**
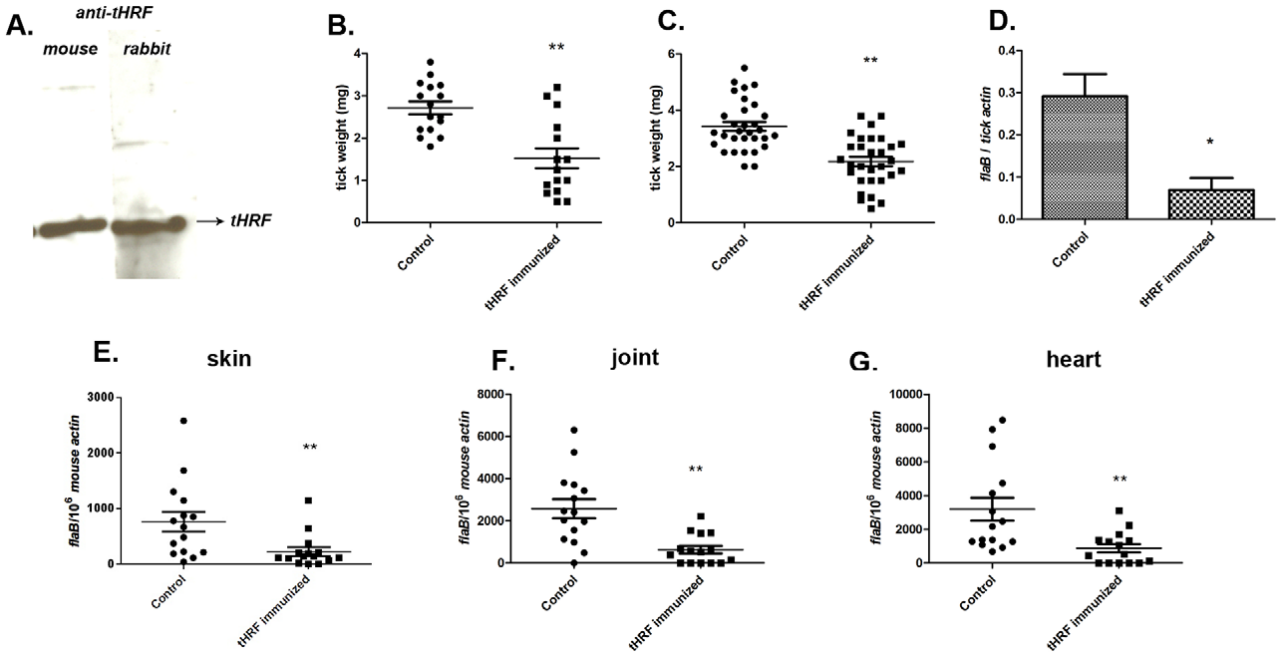
Tick feeding and *B. burgdorferi* transmission was significantly impaired in tHRF immunized mice. (A) tHRF probed with sera from mice or rabbits actively immunized with tHRF. (B) Weights of *B. burgdorferi* uninfected or (C) infected tick after feeding on tHRF-immunized, or control, mice. (D) Spirochete burden in whole *I. scapularis* after feeding on tHRF-immunized, or control, mice. (E) *B. burgdorferi* burden in murine skin at day 7 post-tick feeding. Spirochete burden in the (F) joints and (G) heart at day 21 post-tick feeding. Horizontal lines and bars represent the mean values ± the SEM. * *p*<0.05 and ** *p*<0.01, when compared with the control group. Results are pooled from 3 independent experiments.

We then assessed the ability of the ticks to feed on mice actively immunized with tHRF. Group of 5 mice were immunized with recombinant tHRF, or adjuvant (control). Immunoblots confirmed that mice generated antibodies against tHRF following active immunization (Figure 4A). The tick weights were significantly decreased when *B. burgdorferi*-infected, or uninfected, nymphs fed on tHRF immunized mice compared to ticks that fed on control mice (Figure 4, B and C). The spirochete load was also markedly reduced in ticks fed on tHRF-immunized mice (Figure 4D) and in murine skin (at day 7 post-infection) (Figure 4E) and in joints and hearts (at 3 weeks post-infection) in the tHRF-immunized group compared to that in control mice (Figure 4, F–G). 20–33% of tHRF immunized mice (N = 15) were PCR negative, while 100% of the mice in the control groups (N = 15) were PCR positive for *B. burgdorferi flaB* amplicon (Figure 4, E–G).

Our above experiments focused on 72 h post tick attachment- a specific time point at which 30–40% of the ticks from the control groups successfully complete engorgement and drop off the mice, and the remaining ticks nearing engorgement. To address the role of tHRF on 72–96h post tick attachment, all the ticks were allowed to feed to repletion on tHRF antiserum immunized mice or control mice. While, ticks in the control group fed to repletion and detached around 72–84 h of attachment, ticks fed on tHRF-immunized animals fed to repletion around 96 h after attachment. Further, 10–20% of ticks from the tHRF group remained attached to the mouse even after 96 h, (Figure S1A). The engorgement weights of ticks fed on tHRF-antiserum immunized mice were also significantly less than the engorgement weights of ticks fed on control mice (Figure S1B), consistent with our data obtained from 72 h fed ticks (Figure 2–4).

### tHRF binds to basophils and stimulates histamine release

**Figure 5:**
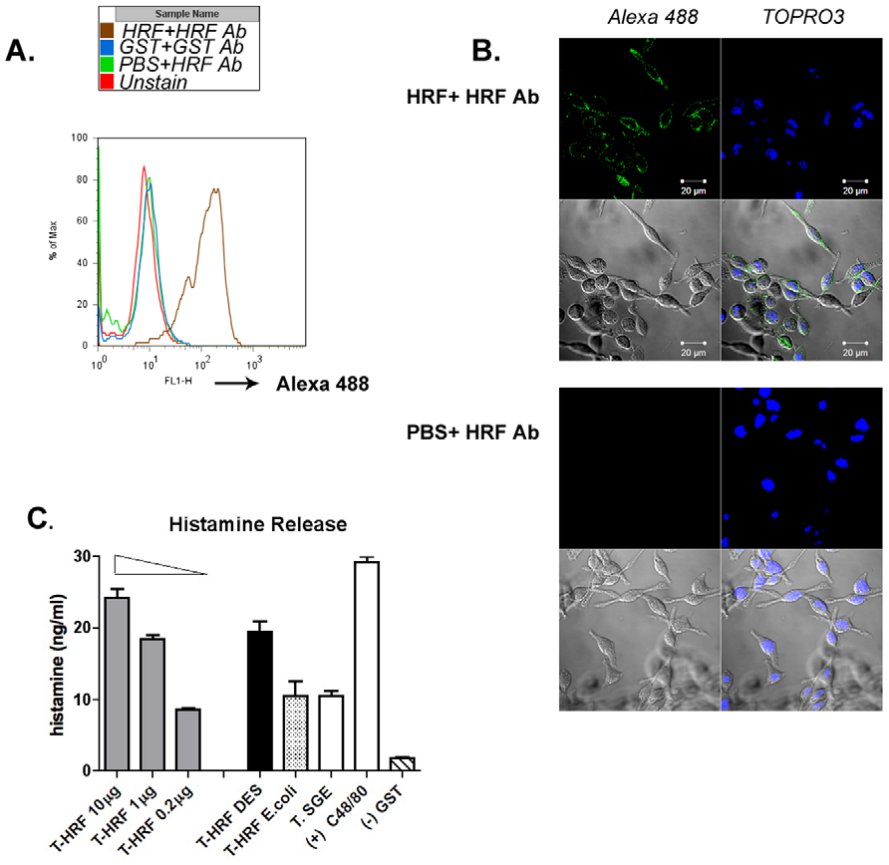
tHRF binds to basophils and stimulates histamine release. (A) Flow cytometric analysis of recombinant tHRF binding to basophils, – as defined by the shift in the FL1 (green) channel when compared to the controls. (B) Confocal imaging of recombinant tHRF binding to the surface of basophils (tHRF+ Alexa 488 labeled tHRF Ab). and Negative control (PBS+Alexa 488 labeled tHRF Ab). Nuclei were counterstained with TOPRO3. (C) Recombinant tHRF (generated from Drosophila (DES) or *E. coli*) and extract from nymphal tick salivary gland (T. SGE) stimulate histamine release from basophils. Recombinant tHRF (DES) induces histamine release in a dose-dependent manner. Recombinant GST used as a negative control; Substance C48/80, a calcium ionophore shown to induce histamine release in basophils [22] served as a positive control. Results are expressed as the mean ± the SEM. Representative results from at least 3 independent experiments were shown.

Mammalian histamine release factor binds to basophils and stimulates histamine release [24]. Since tHRF shares substantial homology with mammalian histamine release factors, we postulated that tHRF might also adhere to host basophils and induce histamine secretion. An *in vitro* binding assay was performed using a rat basophil cell line and recombinant tHRF (tHRF shares 57% similarity with rat HRF at amino acid level, and rat HRF is 100% identical to mouse HRF). Flow cytometry and confocal imaging showed that recombinant tHRF bound to rat basophils (Figure 5, A and B). To examine the influence of tHRF on histamine release, basophils were incubated with recombinant tHRF, or nymphal tick salivary gland extracts (T.SGE). Recombinant tHRF and tick salivary gland extracts stimulated histamine release from basophils (Figure 5C).

### tHRF is required for tick rapid feeding

**Figure 6:**
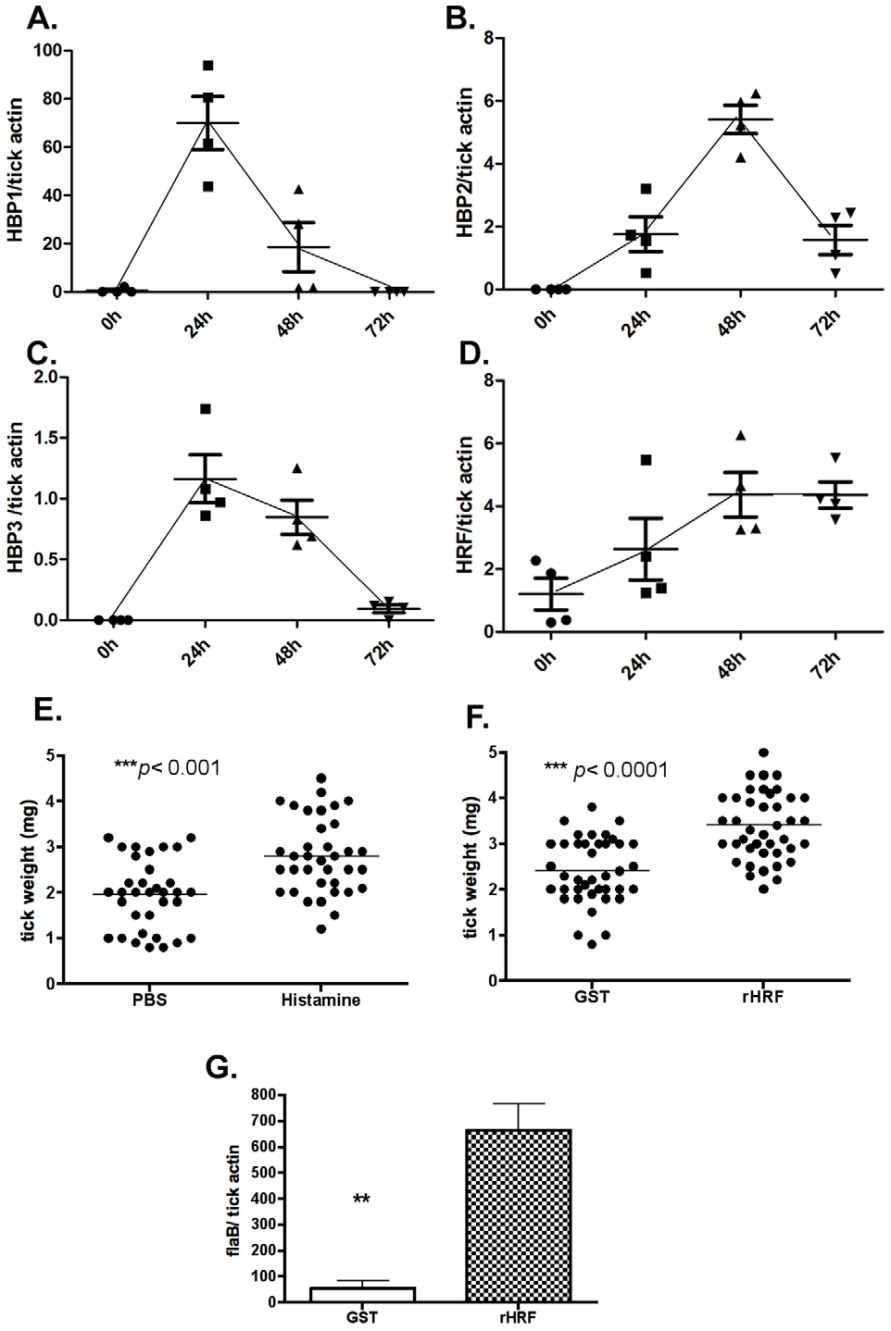
tHRF is required for tick rapid feeding. Quantitative RT-PCR assessment of the expression pattern of: (A) tick histamine binding protein 1 (*HBP1*), (B) *HBP2*, (C) *HBP3* and (D) tick histamine release factor (*tHRF*). (E) Weights of ticks at 72 h post tick attachment on mice treated with histamine (10mM) or PBS (control). (F) Weights of ticks at 72 h post tick attachment on mice treated with recombinant tHRF (10µg) or GST (control). (G) *B. burgdorferi* burden in ticks fed on mice treated with recombinant tHRF (10µg) or GST (control). Horizontal lines and bars represent the mean values ± the SEM. * *p*<0.05 and ** *p*<0.01. Representative results from at least 3 independent experiments were shown.

Ticks are sensitive to histamine during the early stage of blood feeding, and express histamine binding proteins to counteract this effect. However, tick sensitivity to histamine wanes after 3 days of attachment to a host [20]. Quantitative RT-PCR analysis showed preferential expressions of 3 histamine binding proteins in the salivary glands of *I. scapularis* nymphs at 24–48 h post tick attachment (Figure 6A–C). However, tHRF was preferentially expressed at 48–72 h post tick attachment (Figure 6 D). Since tHRF induces histamine release, histamine might play an under-appreciated role in the late/rapid phase of tick feeding. To confirm this, histamine or recombinant tHRF was injected into the skin - at the *I. scapularis* bite site - 60 h after tick-attachment. The tick weights at 72 h were significantly increased when *I. scapularis* nymphs fed on mice given histamine or recombinant tHRF compared to ticks fed on control mice (Figure 6, E and F). The *B. burgdorferi* burden was also higher in ticks fed on tHRF-treated mice compared to ticks that fed on control mice (Figure 6G).

## Discussion

The incidence of tick-borne diseases has steadily increased over the past few years, and effective vaccines against most tick-borne pathogens are not currently available [4]. *I. scapularis* is the major vector of Lyme disease in the USA [2], [15]. Further, *I scapularis* can serve as efficient vectors of *A. phagocytophilum*, *B. microti*, and Powassan virus (a tick-borne encephalitis causing virus). The last decade has seen an increased functional understanding of tick salivary proteins and their critical interactions with the host and pathogen [17], [18], [25]. This information has also offered a new approach to develop effective vaccines against ticks and the pathogens they transmit by simultaneously targeting the pathogen and the tick [26].

The identification of tick proteins potentially involved in pathogen transmission is an important step in the development of effective tick vaccines [1], [18]. The presence of *B. burgdorfer*i within ticks may alter the expression level of selected genes that encode antigens in saliva [19], [27]. One of best characterized genes is *salp15* [27]; our recent study suggests that immunization with Salp15 could reduce the transmission of *B. burgdorferi* from infected ticks to mice, although Salp15 antibodies did not influence the ability of ticks to feed. The action is mainly due to the interaction between Salp15 antibody, Salp15 and *Borrelia* [26].

We performed a 2DIGE analysis to identify additional tick salivary proteins modulated by spirochetes. We found that tHRF was up-regulated in *Borrelia*-infected tick salivary glands. HRF is an evolutionally conserved multiple-function protein [28], also a novel cytokine that provokes the release of histamine by both IgE-dependent and IgE-independent mechanisms from mammalian basophils and mast cells [29]. In addition to mammalian HRF, HRF homologs have also been identified in *Plasmodium falciparum* parasite [30], *Dermacentor variabilis* [22], [31], [32] and *Dermanyssus gallinae* [33]. The latter study further indicated that antibodies against HRF increased the mortality of the mites after engorgement, suggesting its potential as a vaccine antigen [33].

Histamine, secreted by basophils in blood and mast cells in tissues, plays a deleterious role during tick feeding. Histamine is a mediator of the itch response and promotes the recruitment of pro-inflammatory cells to the tick bite site – and these immune response prevent tick attachment to the skin of the host [20], [34]. However, the Ixodes tick encodes several histamine binding proteins (HBPs) to counteract the effect of histamine [21], [35]. The elaboration of a histamine release factor in tick salivary glands therefore seemed counterintuitive, since such an activity would be detrimental to tick feeding. Mulenga et al. [22] suggested that ticks might need either HBPs or HRF, depending on its feeding phase. Tick feeding involves a complex series of 9 sequential stages [2]. Host seeking and engagement with the host precede actual tick attachment and establishment of the feeding lesion. The early phase of tick feeding that lasts about 24 h post attachment is sensitive to histamine [20]. We observed increased expression of Histamine Binding Proteins (*HBP*) in *I. scapularis* nymphal salivary glands during this early phase of tick feeding and might be critical to counter the effect of histamine (Figure 6). Ticks imbibe very little blood during this early phase of feeding. About 60–72h post tick attachment, which includes the rapid feeding phase, tick sensitivity to histamine significantly declines [20], [34], and ticks fully engorge. During this phase the expressions of HBPs appear to be significantly decreased, and the expression of tHRF increases (Figure 6). We speculate that this reciprocal expression of HBPs and tHRF might help increase the local concentration of histamine at the tick-feeding site during the rapid feeding phase. Increased histamine concentration might modulate the vascular permeability to enhance blood flow into the tick feeding site and facilitate tick engorgement.

*B. burgdorferi* replicate after the tick begins to take a blood meal, and transmission to the host begins about 36–48 h post tick attachment, a time coincident with active spirochete replication and migration to the salivary glands [14], [36]. Temperature and host blood are critical signals for *B. burgdorferi* replication and dissemination from the midgut. Since the feeding ability of the tHRF-deficient ticks was significantly impaired, the replication and dissemination of *Borrelia* inside the ticks was also significantly decreased (Figure 2C). Consequently, the spirochete transmission from tick to mouse was also reduced (Figure 2D), and 20–30% of the mice immunized with tHRF were fully protected from *Borrelia* infection based on the absence of a detectable *flaB* signal in Q-PCR (Figure 3 and 4). It is also conceivable that the vasodilatory effect of histamine, might additionally contribute to the efficient dissemination of *Borrelia* from the original tick-feeding site, where they are deposited, to distal sites.

In summary, for the first time, we demonstrate that the *I. scapularis* salivary protein tHRF is critical for the tick engorgement, and consequently also facilitates *Borrelia* transmission to the murine host. We show that *B. burgdorferi* upregulates the expression of tHRF and immunization with tHRF significantly impairs tick feeding, and decreases *B. burgdorferi* burden in mice. Importantly, these observations underscore the dynamic nature of the temporal interactions between the vector, the host and the pathogen. While vaccine targeting of tHRF alone might not be sufficient to thwart tick feeding and spirochete transmission, blocking tHRF might offer a viable strategy to complement ongoing efforts to develop vaccines to block tick feeding and transmission of tick-borne pathogens.

## Materials and Methods

### *B. burgdorferi*, ticks, and mice

An infectious and low passage isolate of *B. burgdorferi* N40 was used to generate *B. burgdorferi*-infected ticks. Larval, nymphal, and adult *I. scapularis* were maintained in our laboratory. Clean larvae were fed either on naïve C3H mice to generate naïve nymphs or on *B. burgdorferi*-infected C3H mice to generate infected nymphs. Female C3H/HeJ (C3H) mice, 4 to 6 weeks of age, were obtained from the Jackson Laboratory.

### Ethics statement

Animals were housed and handled under the Guide for the Care and Use of Laboratory Animals of the National Institutes of Health. The animal experimental protocol was approved by the Yale University's Institutional Animal Care & Use Committee (Protocol Permit Number: 2008-07941). All animal infection experiments were performed in a Bio-safety Level 2 animal facility, according to the regulations of Yale University.

### 2D fluorescence differential gel electrophoresis (DIGE) of nymphal salivary gland proteins

A quantitative analysis of the *I. scapularis* salivary gland proteome was carried out by 2D fluorescence differential gel electrophoresis (DIGE) at the W.M. Keck Facility at Yale University. Salivary gland extracts from 200 clean and *Borrelia*-infected *I. scapularis* nymphs fed for 66–72 h were suspended in a cell lysis buffer (7M urea, 2M thiourea, 4% CHAPS, 25 mM Tris, pH 8.6 at 4°C) and equal amounts of protein (50 µg) from *Borrelia*-infected and clean salivary gland extracts were then differentially labeled *in vitro* with Cy3 and Cy5 N-hydroxysuccinimidyl ester dyes as described in the Ettan DIGE manual (GE Healthcare, NJ) and electrophoresis and analysis performed essentially as described earlier [13]. The gel was sequentially scanned using the Typhoon 9410 Imager (GE Healthcare, Piscataway, NJ) and images exported into the DeCyder (GE Healthcare, NJ) software package to assess differentially expressed protein spots. The protein spots that were increased at least 5-fold in *Borrelia*-infected salivary glands were excised for identification. The gel spots of interest were robotically digested using trypsin prior to analysis on an Applied Biosystems 4800 MALDI-Tof/Tof mass spectrometer. The data was analyzed using the Applied Biosystems GPS Explorer software with Mascot analysis against the NCBI nr database, and a combined peptide mass fingerprint/MS/MS search was done. Spots identified with significant MASCOT scores (*P*<0.05) of 79 were tabulated.

### RNAi

Fed-nymph salivary gland cDNA was prepared as described [37] and used as template to amplify cDNA of *tHRF* (GenBank accession no. DQ066335), and *SSRB* (Signal sequence receptor beta, another tick gene used as a control) (GenBank accession no. DQ066202). The primer sequences are indicated in Table S2 (P11, 12 for tHRF; P13, 14 for SSRB). The resultant amplicons were purified and cloned into the *SacII-XhoI* sites of the L4440 double T7 Script II vector [37]. dsRNA complementary to the DNA insert was synthesized by *in vitro* transcription using the Megascript RNAi kit (Ambion, Austin, TX). The dsRNA was purified and quantified spectroscopically. The microinjection of dsRNA was performed as described previously [37]. Briefly, we injected ≈4 nl of dsRNA (1×10^9^ molecules per nl) or buffer alone (MOCK) into the ventral torso of the idiosoma of nymphal *I. scapularis*. The ticks were allowed to rest for 4∼6 hrs before feeding on mice.

### Quantitative PCR

DNA was extracted from mouse tissues and ticks using a DNeasy tissue kit (QIAGEN, Valencia, CA) according to the manufacturer's protocol. The nymphal ticks (unfed or fed for 24, 48, and 72 h) were dissected under the microscope to get the tick salivary gland and midgut. Total RNA was extracted using RNeasy mini spin columns (QIAGEN). RNA was converted into first-strand cDNA using random hexamers and Superscript III reverse transcriptase (Invitrogen, Carlsbad, CA) according to the manufacturer's protocol.

All quantitative PCR (Q-PCR) assays were performed with an iCycler (Bio-Rad Laboratories, Hercules, CA) using gene-specific primers, and IQ SYBR green quantitative PCR system (Bio-Rad) or a Taqman quantitative PCR system (Applied Biosystems, CA) with a program consisting of an initial denaturing step of 3 min at 95°C and 45 amplification cycles consisting of 30 s at 95°C followed by 1 min at 60°C. The gene-specific primers (and probes, for Taqman Q-PCR) used for Q-PCR were indicated in Table S2.

### Recombinant protein, antibody production and western blot

The full open reading frame of *tHRF* was amplified from the tick salivary cDNA library using gene specific primers P15, 16 (Table S2). The PCR product was subcloned into the pGEX-6P2 vector (Invitrogen, CA) and transfected into *E.coli* BL21/DE3 strain for protein expression. The recombinant tHRF was purified by GST sephorose 4B and the GST tag was removed by the precision protease on column according to the manufacturer's protocol.

To make recombinant protein using the Drosophila S2 cell system, the full open reading frame of *tHRF* was subcloned into pMT/BiP/V5-His A vector (Invitrogen) using primers P17, 18 (Table S2) and transfected into Drosophila S2 cells (Invitrogen, CA) in combination with the hygromycin selection vector pCOHYGRO for stable transfection. The stable transformants were selected using 300 µg/ml hygromycin-B for 3–4 weeks. The recombinant tHRF with 6-His tag were induced and purified with Talon affinity column as described previously [38].

To generate polyclonal antisera, tHRF (without the GST tag) produced in *E. coli* was emulsified in complete Freund's adjuvant and injected into groups of 2–3 rabbits (100 µg/animal). The animals were boosted twice at 3-week intervals with the same dose of antigen in incomplete Freund's adjuvant, and the sera were collected 2 week after the second boost.

A western blot was performed to analyze the protein expression of tHRF in adult tick saliva, nymphal salivary gland extract, midgut extract and whole nymphs. The tick saliva and tissue extract were prepared as described [22]. Protein preparations were separated on a 4–15% gradient poly-acrylaminde gel and transferred on to a PVDF membrane. The membranes were probed with polyclonal anti-tHRF antibody followed by HRP-conjugated anti-Rabbit IgG and detected with enhanced luminol-based detection (ECL) kit (GE bioscience).

### Passive and active immunization

Groups of 5 mice were passively immunized with 200 µl of normal rabbit serum, anti-Salp25D antiserum (as controls) or anti-tHRF antiserum, respectively (Salp25D is a tick salivary protein that does not influence tick feeding [23]). 24 h after immunization, 6 *B. burgdorferi*-infected nymphal ticks or 10 non-infected nymphs were placed on each mouse. After 72h, the ticks were collected and weighed to analyze the feeding efficiency. For the *B. burgdorferi*-infected tick experiment, the *Borrelia* burden in ticks as well as in the localized skin specimen at 7 post tick repletion and in the murine heart and joints at 3 weeks post-infection were determined by measuring *flaB* copies using quantitative PCR.

To address the role of tHRF on 72–96 h post tick attachment, all the ticks were allowed to feed to repletion on tHRF antiserum immunized mice or control mice (normal rabbit serum immunized). After 60 h post tick attachment, the mice were examined every 12 h and the number of tick detached from the mice were recorded. The weights of ticks after repletion were measured as described above.

In the active immunization study, groups of 5 mice were immunized by subcutaneously injecting 10 µg of purified recombinant tHRF suspended in complete Freund's adjuvant, or adjuvant alone (mock control). Mice were boosted with 5 µg of antigen suspended in incomplete Freund's adjuvant every two weeks. Before tick challenge, mice were bled and the anti-tHRF antibody titer was analyzed by western blot. The tick challenge and pathogen burden analysis were performed using the same methods described above.

### Binding and histamine release assay

To test whether tHRF binds to mammalian basophils, an *in vitro* binding assay was performed as described previously [24]. Briefly, a rat basophilic leukemia cell line RBL-2H3 was purchased from American type culture collection (ATCC, Manassas, VA). Cells were cultured to confluence in a 6-well plate and then incubated with recombinant tHRF (generated from *E. coli*) or GST in 1% FCS or buffer alone at 4°C for 2hrs. After 3 washes with PBST (PBS+ 0.1% Tween 20), the cells were incubated with purified Alexa 488 labeled anti-tHRF IgG or Alexa 488-anti-GST IgG in 1% FCS plus 1% rat isotype IgG buffer. The IgG labeling was performed using the Alex488 easy labeling kit (Invitrogen, CA) according to manufacture's direction. After 3 washes with PBST, the cells were fixed with 4% PFA and permeablized with 1% Triton X-100, and the nuclei were stained with TOPRO3. The cells were then analyzed by microscopy and Flow Cytometry.

To investigate whether tHRF can induce histamine secretion from basophils, a histamine release assay was performed using the method described [22]. The possible endotoxins from all test protein preparations were eliminated by passing the protein preparations through endotoxin-free columns (PIERCE, Rockford, IL). Varying concentrations of 5, 0.5 and 0.1 µg ml^−1^ of endotoxin-free HRF (DES) or GST (as negative control) were added to confluent RBL-2H3 cells (in 2 ml media) and incubated at 37°C for 30 min. To determine whether, native tHRF in tick tissue extracts could also induce histamine release, 1.0 µg ml^−1^ nymphal tick salivary gland extracts were assayed for histamine release. Substance C48/80 (Sigma, St Louis, MO), a calcium ionophore was used at 0.5 µg ml^−1^ for positive control. A histamine ELISA kit purchased from Research diagnostic Inc. (Flanders, NJ) was used to determine histamine concentrations in culture supernatants.

### Effect of histamine or tHRF on tick rapid feeding

To investigate the role of histamine and tHRF on late stage of tick feeding, *B. burgdorferi* infected nymphal ticks were fed on naïve mice for 60h. Then 10mM of histamine or 10 µg of recombinant tHRF were injected into the mouse skin at the tick bite site (usually around the ear). Control mice were given the same amount of PBS or recombinant GST. At 72h post-tick attachment, the tick weights were measured and *B. burgdorferi* burden in ticks were analyzed by Q-PCR.

### Statistics

Results are expressed as the mean ± the SEM. The significance of the difference between the mean values of the groups was evaluated by Student's *t* test with StatView software (SAS Institute).

### Gene accession numbers

The GenBank accession numbers for the genes related with this study: tHRF/DQ066335; SSRB/DQ066202; Salp25D/AF209911; HBP1/DQ066014; HBP2/DQ066128; HBP3/DQ066002.

## Supporting Information

Figure S1 tHRF antiserum significantly delays tick feeding. A) Assessment of percentage of replete ticks at different time points post tick attachment on immunized mice. (NRS: normal rabbit serum). Results are expressed as the mean + the SEM. B) The weights of replete ticks. Horizontal lines and bars represent the mean values ± the SEM. * *p*<0.05. Representative results from at least 3 independent experiments were shown.
(0.57 MB TIF)

Table S1 2D Fluorescence Differential Gel Electrophoresis (DIGE) analysis and identification of proteins with increased expression in *Borrelia burgdorferi*-infected nymphal salivary glands by Matrix-Assisted Laser Desorption/Ionization.
(0.03 MB DOC)

Table S2 Oligonucleotide primers and probes.
(0.05 MB DOC)
