# Supplementary figures and images for: Tick Histamine Release Factor Is Critical for Ixodes scapularis Engorgement and Transmission of the Lyme Disease Agent
Source: PLoS Pathog. 2010 Nov 24;6(11):e1001205. doi: 10.1371/journal.ppat.1001205 (PMC2991271; doi:10.1371/journal.ppat.1001205)

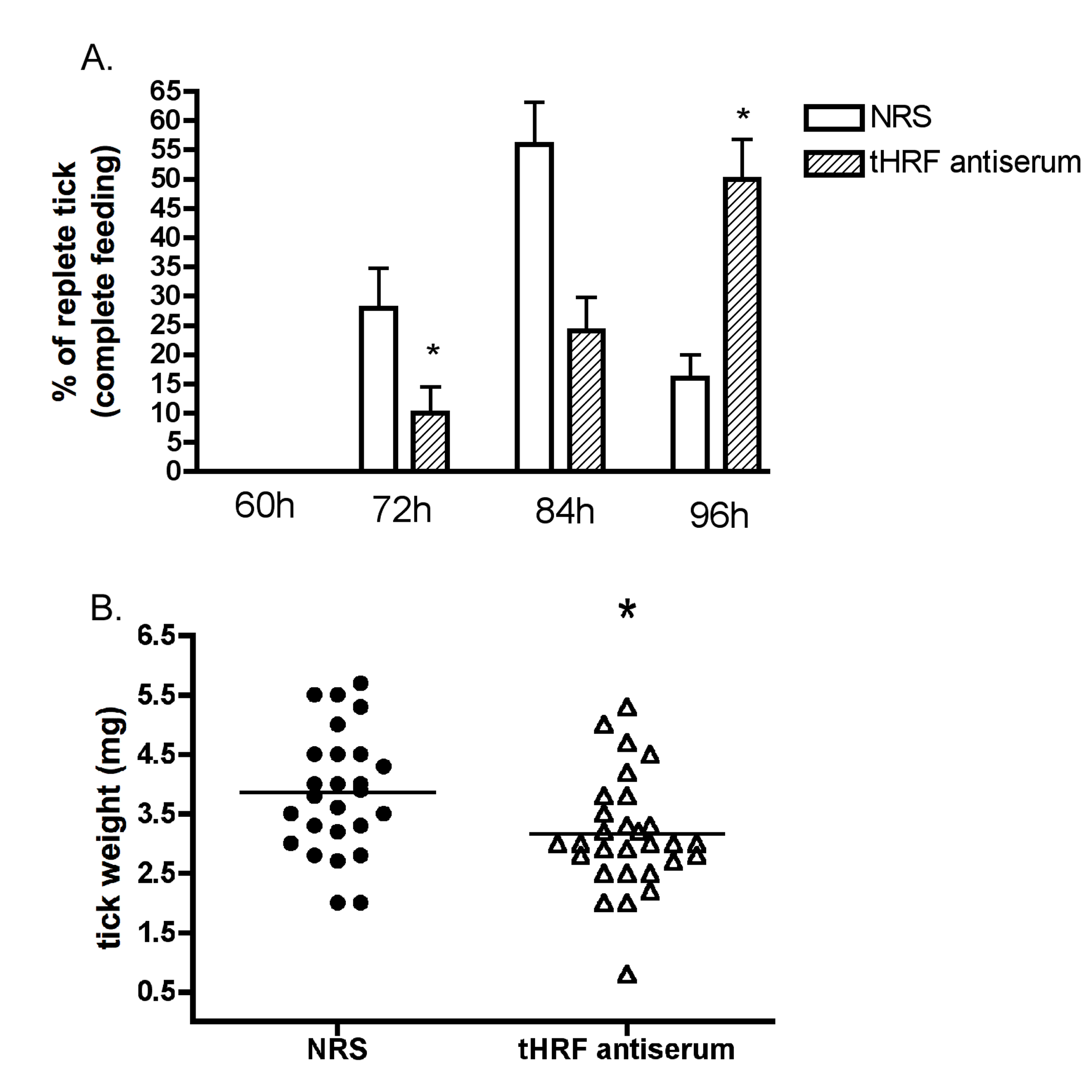

Supplement: Figure S1 — tHRF antiserum significantly delays tick feeding. A) Assessment of percentage of replete ticks at different time points post tick attachment on immunized mice. (NRS: normal rabbit serum). Results are expressed as the mean + the SEM. B) The weights of replete ticks. Horizontal lines and bars represent the mean values ± the SEM. * p<0.05. Representative results from at least 3 independent experiments were shown. (0.57 MB TIF) [file ppat.1001205.s001.tif]
