# Supplementary material for: Tick Histamine Release Factor Is Critical for Ixodes scapularis Engorgement and Transmission of the Lyme Disease Agent
Source: PLoS Pathog. 2010 Nov 24;6(11):e1001205. doi: 10.1371/journal.ppat.1001205 (PMC2991271; doi:10.1371/journal.ppat.1001205)
Supplement: Table S1 — 2D Fluorescence Differential Gel Electrophoresis (DIGE) analysis and identification of proteins with increased expression in Borrelia burgdorferi-infected nymphal salivary glands by Matrix-Assisted Laser Desorption/Ionization. (0.03 MB DOC) [file ppat.1001205.s002.doc]

**Table S1.** 2D Fluorescence Differential Gel Electrophoresis (DIGE) analysis and identification of proteins with increased expression in *Borrelia burgdorferi*-infected nymphal salivary glands by **M**atrix-**A**ssisted **L**aser **D**esorption/**I**onization.

| **Protein ID/Accession #** | **Protein name** | **Fold-change in expression**  ***(Borrelia*-infectedSG/Clean SG)** | **Numbers of Peptides identified** | **% Coverage**  **of the Protein** |
| --- | --- | --- | --- | --- |
| Q4PLZ3* | Translationally- controlled tumor protein/ Histamine release factor | 7 | 11 | 72 |
| XP_002414113 | Signal sequence receptor beta | 10 | 5 | 37 |
| XP_00243444 | Serpin 2 precursor protein | 7 | 7 | 24 |
| AAY66973 | Protein disulphide isomerase | 5 | 12 | 47 |

* The asterisk indicates the protein characterized in this study.
