# Supplementary material for: Tick Histamine Release Factor Is Critical for Ixodes scapularis Engorgement and Transmission of the Lyme Disease Agent
Source: PLoS Pathog. 2010 Nov 24;6(11):e1001205. doi: 10.1371/journal.ppat.1001205 (PMC2991271; doi:10.1371/journal.ppat.1001205)
Supplement: Table S2 — Oligonucleotide primers and probes. (0.05 MB DOC) [file ppat.1001205.s003.doc]

Table S2. Oligonucleotide primers and probes

| Number | Sequence (5’-3’) | Purpose |
| --- | --- | --- |
| P1 | AGCTGAAGAGCTTGGAATGC | Forward primer for Quantitative-PCR of *Borrelia burgdorferi* *flaB* |
| P2 | TTGGTTTGCTCCAACATGAA | Reverse primer for Quantitative-PCR of *Borrelia burgdorferi flab* |
| *Probe 1* | TCCAAGACGCTTGAGACCCTGAAA | Probe for Quantitative-PCR of *Borrelia burgdorferi flaB* |
| P3 | GATCATGTTCGAGACCTTCA | Forward primer for Quantitative-PCR of tick beta-actin |
| P4 | CGATACCCGTGGTACGA | Reverse primer for Quantitative-PCR of tick beta-actin |
| *Probe 2* | CCATCCAGGCCGTGCTCTC | Probe for Quantitative-PCR of tick beta-actin |
| P5 | AGAGGGAAATCGTGCGTGAC | Forward primer for Quantitative-PCR of mouse beta-actin |
| P6 | CAATAGTGATGACCTGGCCGT | Reverse primer for Quantitative-PCR of mouse beta-actin |
| *Probe 3* | CACTGCCGCATCCTCTTCCTCCC | Probe for Quantitative-PCR of mouse beta-actin |
| P7 | CCTGGTGGAGACGGGTTTCT | Forward primer for Quantitative-PCR of tick histamine release factor *HRF* |
| P8 | TTGACGGCCTCTGTGAGCTT | Reverse primer for Quantitative-PCR of tick histamine release factor *HRF* |
| P9 | TCTCCTGCCTCTGCCTGTGC | Forward primer for Quantitative-PCR of tick single sequence receptor beta *SSRb* |
| P10 | CGACGATGATGTCTCGCCCT | Reverse primer for Quantitative-PCR of tick single sequence receptor beta *SSRb* |
| P11 | TCC*CCGCGG*GCCCTGATGGACAAGTGGAA | Forward primer for constructing dsRNA plasmid L4440-HRF |
| P12 | CCG*CTCGAG*CAAGAGCCCGTGCTTGAAGA | Reverse primer for constructing dsRNA plasmid L4440-HRF |
| P13 | TCC*CCGCGG*CATCTACAACGTCGGCGGAA | Forward primer for constructing dsRNA plasmid L4440-SSRb |
| P14 | CCG*CTCGAG*GAACCAGAGCAGGAAGGGAA | Reverse primer for constructing dsRNA plasmid L4440-SSRb |
| P15 | cg*GGATCC*atgctgcttttcaaggatatcttgac | Forward primer for constructing plasmid pGEX-6p2-HRF |
| P16 | g*GAATTC*ttactgcttctcttccaagagccc | Reverse primer for constructing plasmid pGEX-6p2-HRF |
| P17 | g*gaattc*tatgctgcttttcaaggatatcttgac | Forward primer for constructing plasmid pMT-HRF |
| P18 | ccg*ctcgag*ctgcttctcttccaagagcc | Reverse primer for constructing plasmid pMT-HRF |
| P19 | AAcacgcaaccggacaat | Forward primer for Quantitative-PCR of tick *HBP1* |
| P20 | ctcggaggtaaacggatgaa | Reverse primer for Quantitative-PCR of tick *HBP1* |
| P21 | ctacgatgctcaaagggagc | Forward primer for Quantitative-PCR of tick *HBP2* |
| P22 | gcgcttcttagaccaaatgc | Reverse primer for Quantitative-PCR of tick *HBP2* |
| P23 | CTTGCTGTCGCTTCTCGTTATT | Forward primer for Quantitative-PCR of tick *HBP3* |
| P24 | AAAGTTTTCCGGGTCATCCTCT | Reverse primer for Quantitative-PCR of tick *HBP3* |
